# *In vivo* Functional Characterization of Hydrophilic X2 Modules in the Cellulosomal Scaffolding Protein

**DOI:** 10.3389/fmicb.2022.861549

**Published:** 2022-04-07

**Authors:** Xuanyu Tao, Jiantao Liu, Megan L. Kempher, Tao Xu, Jizhong Zhou

**Affiliations:** ^1^Institute for Environmental Genomics and Department of Microbiology and Plant Biology, University of Oklahoma, Norman, OK, United States; ^2^School of Life Sciences, Jiangxi Science & Technology Normal University, Nanchang, China; ^3^Section on Pathophysiology and Molecular Pharmacology, Joslin Diabetes Center, Boston, MA, United States; ^4^Department of Microbiology and Immunobiology, Harvard Medical School, Boston, MA, United States; ^5^Earth Sciences Division, Lawrence Berkeley National Laboratory, Berkeley, CA, United States

**Keywords:** cellulosome, X2 module, cellulose degradation, *Clostridium cellulolyticum*, motif deletion

## Abstract

As part of free cellulases or scaffolding proteins in cellulosomes, the hydrophilic non-catalytic X2 module is widely distributed in cellulolytic *Clostridia* or other *Firmicutes* bacteria. Previous biochemical studies suggest that X2 modules might increase the solubility and substrate binding affinity of X2-bearing proteins. However, their *in vivo* biological functions remain elusive. Here we employed CRISPR-Cas9 editing to genetically modify X2 modules by deleting the conserved motif (NGNT) from the CipC scaffoldin. Both single and double X2 mutants (X2-N: near the N terminus of CipC; X2-C: near the C terminus of CipC) presented similar stoichiometric compositions in isolated cellulosomes as the wildtype strain (WT). These X2 mutants had an elongated adaptation stage during growth on cellulose compared to cellobiose. Compared to WT, the double mutant ΔX2-NC reduced cellulose degradation by 15% and the amount of released soluble sugars by 63%. Since single X2 mutants did not present such obvious physiological changes as ΔX2-NC, there seems to be a functional redundancy between X2 modules in CipC. The *in vivo* adhesion assay revealed that ΔX2-NC decreased cell attachment to cellulose by 70% but a weaker effect was also overserved in single X2 mutants. These results highlight the *in vivo* biological role of X2 in increasing cellulose degradation efficiency by enhancing the binding affinity between cells and cellulose, which provides new perspectives for microbial engineering.

## Introduction

As a sustainable and carbon-neutral energy source, biofuels are one of the best alternative energy forms for replacing fossil fuels (Farrell et al., 2006; Naik et al., 2010). Compared to other biomass resources including food grade sources, municipal solid waste, or algae, non-edible lignocellulosic feedstock is a promising source of material for biofuel production as it is cheap, abundant and renewable (Naik et al., 2010; Liao et al., 2016). Although there are many platforms for bioconversion of lignocellulose into biofuels, such as non-isothermal simultaneous saccharification and simultaneous saccharification and co-fermentation, the common barrier for these bioconversion processes is the high cost for cellulase production (Balan, 2014; Liao et al., 2016; Lynd, 2017). In order to reduce the cost of cellulase production and increase the efficiency of bioconversion of lignocellulose, the on-site saccharification strategies, which include consolidated bioprocessing (CBP) and consolidated bio-saccharification (CBS), have been proposed (Lynd et al., 2002, 2005; Liu et al., 2020).

As a model mesophilic clostridial species, *Clostridium cellulolyticum* can hydrolyze cellulose or hemicellulose and then ferment hydrolysis products to ethanol and other organic acids, which enables it as a potential candidate strain for CBP (Desvaux, 2005). Like other cellulolytic clostridia, *C. cellulolyticum* possesses an extracellular enzymatic complex termed the cellulosome, which allows *C. cellulolyticum* to efficiently degrade crystalline cellulose and enables it as a potential candidate strain for CBS (Gal et al., 1997; Shoham et al., 1999; Liu et al., 2020). The pivotal component of the cellulosome in *C. cellulolyticum* is a scaffolding protein/integrating protein encoded by the *cipC* gene, to which up to eight different catalytic cellulases can bind. Without catalytic activity, the CipC scaffoldin contains eight Cohesion modules (type I), one carbohydrate binding module (CBM) and two X2 modules (Supplementary Figure 1; Gal et al., 1997; Schwarz, 2001). The Cohesion module allows the binding from cellulases with type I dockerin (Fierobe et al., 1999). The CBM, belonging to the family IIIa (CBM3a), carries out binding between the entire cellulosome and cellulosic substrate (Boraston et al., 2004). The X2 modules are two hydrophilic domains, each of which contains 100 amino acid residues (Mosbah et al., 2000). In addition to being a part of the CipC scaffoldin, the X2 module is also found in other scaffoldin proteins from other cellulosome-producing bacteria, such as *Clostridium thermocellum* (Lamed and Bayer, 1988), *Clostridium cellulovorans* (Doi et al., 1994), and even in free cellulase enzymes from non-clostridial cellulolytic bacteria, such as *Lachnoclostridium phytofermentans* (Ravachol et al., 2015; Vita et al., 2019) and *Paenibacillus polymyxa* (Pasari et al., 2017). This suggests that X2 modules are widely distributed and may serve an important role in the biodegradation of lignocellulosic biomass. Based on the structure and *in vitro* biochemistry assays of X2 modules, it belongs to the immunoglobulin superfamily and has been predicted to be associated with the localization of the cellulosome, cellulose binding, cell wall binding, or increased enzymatic activities for free cellulase (Kosugi et al., 2004; Chanal et al., 2011; Ravachol et al., 2015; Pasari et al., 2017; Zhang et al., 2018; Tarraran et al., 2021). However, little is known about its *in vivo* biological functions and significance.

It is technically challenging to determine the *in vivo* significance of non-catalytic X2 modules in multi-modular proteins. With the development of CRISPR-Cas9 nickase-based genome editing method (Xu et al., 2015), we are now able to manipulate genes for protein module engineering. Here, using *C. cellulolyticum* as a model strain, we created genetically modified X2 modules in the cellulosomal scaffoldin CipC to study the *in vivo* biological function of X2 modules. We systematically characterized single and double X2 mutants from transcriptional, physiological, and biochemical aspects. Our results demonstrated that the conserved motif in X2 modules is important to cell growth on cellulose and cellulose hydrolysis, probably *via* mediating the binding affinity of cells to cellulose.

## Materials and Methods

### Bacterial Strains and Plasmid Construction

**FIGURE 1:**
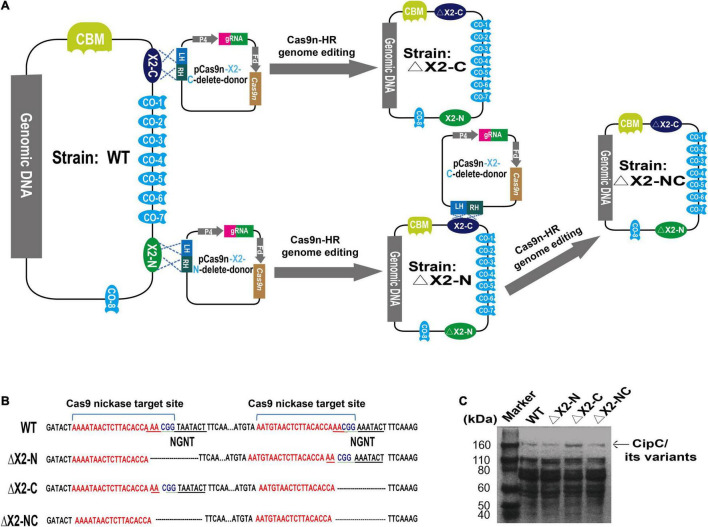
Precise deletion of the conserved motif from the X2 modules to maintain the structural integrity of the CipC protein. **(A)** An overview of the strategy for constructing the dual X2 module mutant by the Cas9 nickase-based genome editing. Both plasmids pCas9n-X2-C-delete-donor and pCas9n-X2-N-delete-donor were used for the ΔX2-NC mutant construction. LH, left homologous; RH, right homologous; P4, P4 promoter (Xu et al., 2015); Fd, ferredoxin promoter (Xu et al., 2015); CBM, carbohydrate binding module; CO, Cohesin. **(B)** DNA sequence showing the deletion of conserved motif from X2 modules in the *cipC* gene. **(C)** SDS-PAGE analysis of cellulosomes extracted from WT and all mutant strains (15 μg protein/lane).

Strains and plasmids used in this study are listed in Table 1. The ΔX2-N and ΔX2-C mutants with deletions of the conserved motif in the N-terminal and C-terminal X2 respectively, were constructed in our previous study (Xu et al., 2015). The dual X2 modules mutant (ΔX2-NC) was constructed using the pCas9n-X2-N-delete-donor to further delete the conserved motif of the X2-N module in ΔX2-C (Li et al., 2012). In brief, for each X2 module, its corresponding variant was created by deleting the 12-bp DNA sequence coding for the conserved Asn-Gly-Asn-Thr motif. To do so, the 23-bp target site for Cas9 nickase was partially overlapped with the deletion region such that the customized donor template could direct the nick repair in the genome to make intended changes (Figure 1A). Finally, the N-terminal and C-terminal X2 modules (X2-N and X2-C) were mutated precisely in the chromosome, generating ΔX2-N and ΔX2-C variants respectively (Xu et al., 2015). Sequential mutation of the N-terminal X2 module in ΔX2-C variant created a dual mutated X2 variant, named ΔX2-NC (Figure 1A). All mutations were verified by amplicon sequencing (Figure 1B).

**TABLE 1:** List of plasmids and strains used in this study.

Strain or plasmid	Phenotype or genotype	Source or References
**Strains**		
Wild type of *C. cellulolyticum* H10	ATCC 35319	Boraston et al., 2004
ΔX2-N	Deletion of the conserved motif (NGNT) of X2-N	Xu et al., 2015
ΔX2-C	Deletion of the conserved motif (NGNT) in X2-C	This study
ΔX2-NC	Deletion of the conserved motif (NGNT) in both X2-C and X2-N	This study
**Plasmids**		
pFdCas9n-p4-pyrF w/2kbΔ	Cmp^r^ in *E.coli* and Tmp^r^ in *C. cellulolyticum* H10	Xu et al., 2015
pMS-RNA	spec^r^ in *E.coli*	Xu et al., 2015
pCR/8w p4-4 prom	spec^r^ in *E.coli*	Xu et al., 2015
pCas9n-X2-N-delete-donor	Cmp^r^ in *E.coli* and Tmp^r^ in *C. cellulolyticum* H10	This study

*E. coli* DH5α strain (Invitrogen, Carlsbad, CA, United States) was used for cloning. Rosetta™(DE3) (Invitrogen, Carlsbad, CA, United States) was used for protein expression. Sequences of X2-C module and CBM3a, amplified from the genome of WT strain, were cloned into pET-28a vectors with a C-terminal 6 × His tag, yielding pET-X2-C and pET- CBM3a, respectively. The pET-ΔX2-C vector was generated from the pET-X2-C by Q5 Site-Directed Mutagenesis Kit (New England Biolabs, Ipswich, MA, United States).

### Media and Culture Conditions

LB medium with 35 μg/ml chloramphenicol or 50 μg/ml kanamycin was used for pCas9n-X2-N-delete-donor cloning or pET-X2C/pET-ΔX2C/pET-CBM3a cloning. Complex modified VM medium supplemented with 2.0 g/L yeast extract was used for reviving and transformation of ΔX2-C (Li et al., 2012). Defined modified VM medium containing necessary vitamin solution and mineral solution was used for growth determination and omics experiments (Higashide et al., 2011). All strains (ΔX2-N, ΔX2-C, ΔX2-NC, and WT) were cultured with 5 g/L cellobiose or 10 g/L Avicel PH101 crystalline cellulose (Sigma-Aldrich, St. Louis, MO, United States) at 34°C anaerobically. Solid VM medium with 1.0% (w/v) of Bacto agar (VWR) and 15 μg/ml thiamphenicol was used for selecting the ΔX2-NC mutant.

### Transformation and Verification of Cellulosome Structure Integrity in Mutant Strains

The pCas9n-X2N-donor was firstly methylated by *Msp*I Methyltransferase (New England Biolabs, Ipswich, MA, United States) and then transformed into ΔX2-C by electroporation as described previously (Li et al., 2012). The pET-X2N, pET-ΔX2N, and pET-CBM3a were transformed into Rosetta™(DE3) according to the manufacturer’s instructions. For each strain, the cellulosome fraction was isolated from 200 ml cellulose-grown culture at mid-exponential growth phase as previously reported (Tao et al., 2020).

### Measurement of Cell Growth and Remaining Cellulose

Microbial growth (ΔX2-N, ΔX2-C, ΔX2-NC, and WT) on cellobiose was determined by optical density (OD) at 600 nm; on cellulose, cell growth was estimated by total protein measurement as previously described (Li et al., 2012). Each strain had three biological replicates. The remaining cellulose and released soluble sugar in the medium were measured by the phenol-sulfuric acid method (Hemme et al., 2011).

### Cell-Cellulose Adhesion Assay

All strains (ΔX2-N, ΔX2-C, ΔX2-NC, and WT) were grown on 5 g/L cellobiose to the same OD_600_. Cells were incubated with or without Whatman filter paper (cellulose) on a tube rotator for 1 h. Then, the OD_600_ of the planktonic phase was determined to reversely infer the binding affinity of cells to cellulose. Each group contained three biological replicates. The relative cell adhesion capability for each strain was normalized to WT.

### Microarray Analysis

All strains (ΔX2-N, ΔX2-C, ΔX2-NC, and WT) were grown in the defined VM medium with 10 g/L cellulose. Each strain was collected at the mid-exponential growth phase. RNA extraction, microarray hybridization, and microarray data analysis were performed as previously described (Tao et al., 2020). For each strain, four biological replicates were performed.

### Expression and Purification of X2-N, ΔX2-N, and CBM3a

For the expression of X2-N or ΔX2-N module, Rosetta™(DE3) containing the corresponding vector was grown to OD_600_ 1.0–1.2 and then induced with 0.5 mM isopropyl-d-1-thiogalactopyranoside (IPTG) for 8 h at 37°C. Then, cells pellets were resuspended in the lysis buffer containing 20 mM Tris-HCl pH 8.0, 100 mM NaCl, 10 mM imidazole. After sonication and centrifugation at 12, 000 *g* for 30 min, the supernatant was loaded to Ni^2+^-nitrilotriacetate affinity resin (Qiagen, Hilden, Germany) equilibrated with 20 mM Tris−HCl pH 8.0, 150 mM NaCl. The X2-N or ΔX2-N protein was eluted with 20 mM Tris−HCl pH 8.0, 150 mM NaCl, 350 mM imidazole and further purified by buffer exchange. For CBM3a expression, Rosetta™(DE3) with pET-CBM3a was grown to OD_600_ 0.6–0.8 and then induced with 0.2 mM IPTG for 20 h at 16°C. The remaining steps were the same as above. The structure of X2-N (PDB: 1EHX) was used as the template to construct the structure of X2-C by homology modeling in SWISS-MODEL^1^.

### The X2 Module-Cell Wall Fragments Binding Assay

A 20 ml of *E. coli* grown in LB medium, *Clostridium thermocellum* and *C. cellulolyticum* grown on 5 g/L cellobiose in VM medium were collected and centrifuged respectively. For each strain, the cell pellets were treated with NaN_3_/Ca^2+^ and washed three times with 50 mM Phosphate-buffered saline (PBS) buffer (Sigma-Aldrich, St. Louis, MO, United States). Then, 50 μg purified X2-N, ΔX2-N, and CBM3a proteins were incubated with collected cell pellets respectively in the PBS buffer at 4°C for 12 h. After centrifugation, the pellet from each incubation was resuspended in the SDS-PAGE Protein Loading Buffer (Thermo Fisher, Waltham, MA, United States) and boiled for 10 min. Finally, the 6x-His Tag antibody (R930-25, Thermo Fisher, Waltham, MA, United States) was used to detect the binding between proteins and cell wall by Western blotting.

### The X2 Module-Cellulose Binding Assay

A 50 μg of X2-N, CBM3a, and BSA (Thermo Fisher, Waltham, MA, United States) were separately incubated with 20 mg Avicel PH101 crystalline cellulose in the reaction buffer (20 mM Tris-HCl pH 8.0) at 4°C for 12 h. After centrifugation, the pellet from each incubation was washed with the reaction buffer three times. Then, the supernatant and pellet were analyzed by SDS-PAGE to detect binding between proteins and cellulose.

### Isothermal Titration Calorimetry Assay

Titration calorimetry measurement was performed with a VP-Isothermal Titration Calorimetry (ITC) calorimeter (MicroCal, Northampton, MA, United States) as previously described (Duff et al., 2011; Brown et al., 2013). The buffer containing 20 mM Tris-HCl pH 8.0 and 100 mM NaCl was used for the assay. In brief, titration with buffer alone (background) and titration from X2-N protein into the buffer (control), were firstly performed as background and control groups. Then, 70 μM X2-N protein was titrated into 10 μM CBM3a protein with an injection volume of 70 μL and constant stirring at 25°C. Finally, a one-site binding model from the Origin 8.0 software (MicroCal) was used for data analysis.

## Results

### Non-catalytic X2 Modules Contain a Highly Conserved Asn-Gly-Asn-Thr Motif

We first evaluated the prevalence of X2 modules (PF03442) in assembled bacterial genomes using HMMER tools^2^ and found 1243 species that genetically encode proteins containing X2 modules including bacteria (1024), eukaryote (214), archaea (4), and unclassified (1). Among bacteria, more than 60% of identified X2 modules were found in the *Firmicutes* phylum including the classes *Bacilli* and *Clostridia*. Interestingly, at the protein level, more than 50% of X2 modules were found in multi-modular cellulases or cellulosomes, adjacent to the CBM module (X2-CBM). These architectural features may indicate an important role of X2 in insoluble carbon-associated microbial metabolism.

**FIGURE 2:**
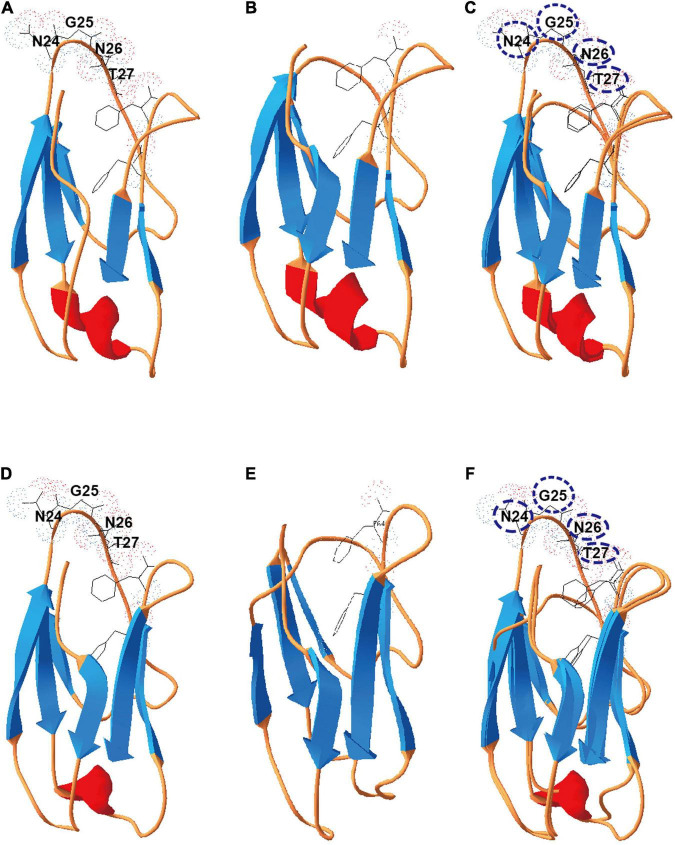
Deletion of the conserved motif (NGNT) may lead to a conformational change of the X2 module. **(A)** The structure of the X2-N module protein; **(B)** the structure of ΔX2-N module in which the conserved NGNT residues were deleted. **(C)** Structures overlapping between X2-N and ΔX2-N modules. **(D)** The structure of the X2-C module protein; **(E)** the structure of ΔX2-C module in which the conserved NGNT residues were deleted; **(F)** structures overlapping between X2-C and ΔX2-C modules.

To test this hypothesis, we selected X2 modules found in the cellulosomal scaffolding protein CipC of *C. cellulolyticum* to interrogate further. These two X2 modules were named as X2-N (near the N terminus of CipC) and X2-C (near the C terminus of CipC) based on their loci in the CipC protein (Supplementary Figure 1). Since a truncated CipC led to the failure of cellulosome assembly (Maamar et al., 2004), a precise genome editing tool is required to mutate the X2 modules without sacrificing the overall architecture of CipC. It is interesting that an alignment of X2-like modules from different bacteria revealed a highly conserved short motif (Asn-Gly-Asn-Thr) (Supplementary Figure 2). This motif is structurally located in an exposed loop on X2-N (PDB: 1EHX) and X2-C (Figure 2). Deletion of this short motif would shorten the loop region and form a wider groove as shown by structure modeling (Figure 2), which would allow for investigation of X2 function in CipC with a minimal impact on CipC structure.

### Single and Dual X2 Modifications in CipC Diminish Physiological Performance on Cellulose

We precisely deleted the conserved motif in the X2 modules of CipC by Cas9 nickase-based genome editing, yielding single (ΔX2-N and ΔX2-C) and double X2 mutants (ΔX2-NC) (Figures 1A,B). SDS-PAGE analysis of cellulosome fractions from cellulose-grown WT and mutant strains demonstrated no significant changes in gel patterns and band intensities (Figure 1C), which suggested the cellulosome architecture was not impeded by the X2 mutations.

**FIGURE 3:**
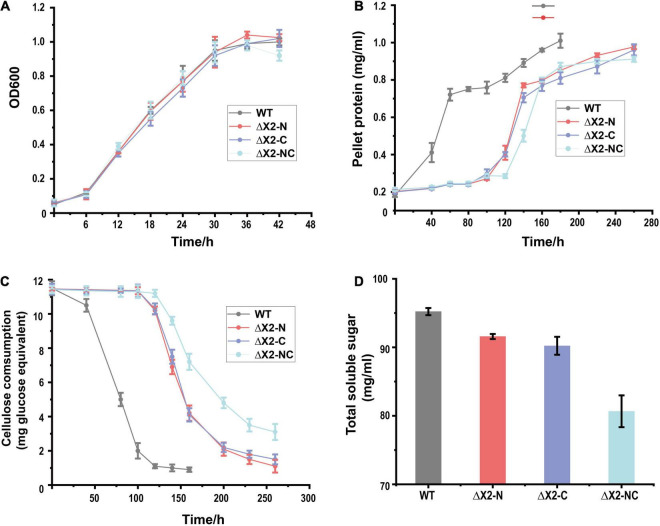
Disruption of X2 modules increased the lag phase and decreased the cellulose degradation efficiency when mutants were grown on cellulose. **(A)** Growth profiles of WT, ΔX2-N, ΔX2-C, and ΔX2-NC grown on cellobiose. **(B)** Growth profiles of WT, ΔX2-N, ΔX2-C, and ΔX2-NC grown on cellulose. **(C)** Cellulose degradation profiles of WT, ΔX2-N, ΔX2-C, and ΔX2-NC. **(D)** Released total soluble sugars in supernatant of medium at final time point for each strain when grown on cellulose. Data are presented as the mean of three biological replicates and error bars represent standard deviation (SD).

To test if X2 was involved in insoluble carbon-dependent microbial metabolism, we monitored the growth of X2 mutants on different carbon sources. On cellobiose, all mutants (ΔX2-N, ΔX2-C, and ΔX2-NC) presented similar growth profiles as WT (Figure 3A). However, when grown on 10 g/L cellulose, ΔX2-N, ΔX2-C, and ΔX2-NC had a much longer adaptation stage than WT before achieving a similar growth rate and final biomass level as WT (Figure 3B), suggesting the importance of X2 modules in cell growth on insoluble cellulose. In addition, compared to WT, the release of soluble sugars in ΔX2-N, ΔX2-C, and ΔX2-NC was decreased by 28, 40, and 63%, respectively. The amount of residual cellulose in ΔX2-NC was 15% higher than WT and approximately 10% higher than ΔX2-N and ΔX2-C. These results concordantly demonstrate that genetic mutation of X2 in CipC can reduce cellulose hydrolysis of the cellulosome (Figures 3C,D and Supplementary Figure 3). This is consistent with previous *in vitro* studies on X2 in *C. cellulovorans* (Kosugi et al., 2004). These data also solidify the importance of the conserved motifs in the X2 module for the first time.

### Disrupted X2 Modules Reduce Cell Adhesion to Cellulose

To understand if a polar effect was introduced to downstream genes or the expressions of other genes were influenced by the X2 mutations (Maamar et al., 2004), we performed microarray-based transcriptomic analysis for the whole transcriptome of *C. cellulolyticum*. We found there were no differentially expressed genes present between WT and X2 mutants grown on cellulose during the exponential phase. Therefore, the disruption of the X2 modules seemed to have influenced cellulose degradation not by regulating gene expressions.

**FIGURE 4:**
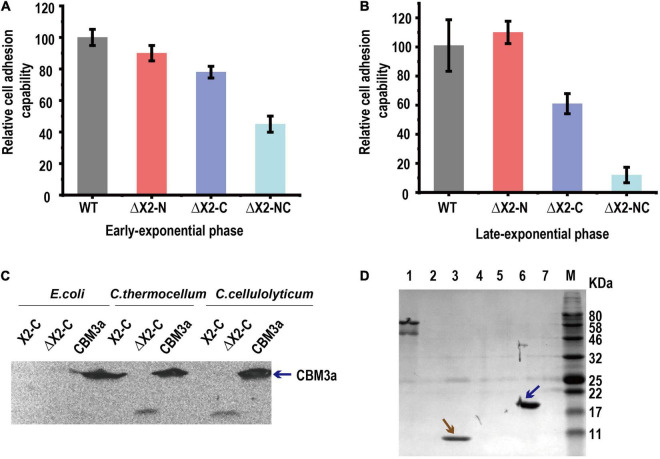
The *in vivo* function of the X2 module was related to the binding affinity between cells and cellulose. Panels **(A,B)**, the relative cell adhesion capability between cells and cellulose for each strain in early exponential **(A)** and late-exponential phase **(B)**. Data are presented as the mean of three biological replicates and error bars represent SD. **(C)** The binding of X2-C, ΔX2-C, and CBM3a proteins to the cell surfaces of *E. coli*, *C. thermocellum*, or *C. cellulolyticum* respectively, determined by western blot. CBM3a was detected in all three strains (as blue arrow indicated), indicating it could bind to the cell surface for both Gram-negative and Gram-positive bacteria. The X2-C could not be detected for any of them, indicating it can not directly bind to the cell surface. The weak band of ΔX2-C was detected in *C. thermocellum* and *C. cellulolyticum*, indicating it had a weak binding affinity with the cell surface of the Gram-positive bacteria. **(D)** Binding of X2-C, CBM3a, and BSA protein to crystalline cellulose, determined by SDS-PAGE. The CBM3a protein and the BSA protein were used as the positive and negative control respectively. The CBM3a was detected in the cellulose pellet (as blue arrow indicated) and X2-C was only detected in the supernatant fraction as same as the BSA negative control (as brown arrow indicated), indicating that X2-C can not directly bind to the cellulose. Lane 1, BSA + Cellulose in supernatant fraction; Lane 2, CBM + Cellulose in supernatant fraction; Lane 3, X2-C + Cellulose in supernatant fraction; Lane 4, blank; Lane 5, BSA + Cellulose in cellulose-containing pellet; Lane 6, CBM + Cellulose in cellulose-containing pellet; Lane 7, X2-C + Cellulose in cellulose-containing pellet.

We then speculated that X2 modules might be key factors responsible for (i) increasing the binding affinity of cellulosomes to cellulose, (ii) increasing the localization and adhesion of cellulosomes to the cell surfaces, or (iii) increasing both binding affinity to cellulose and adhesion to cell surfaces. To determine whether the X2 mutations influenced the binding affinity between cells and cellulose, a cell-cellulose adhesion assay was performed. With cells collected at the early exponential stage, we found ΔX2-NC mutant decreased cell adhesion to cellulose by 50% when compared to WT. The ΔX2-C presented a more profound effect than ΔX2-N (Figure 4A). The decrease in cell adhesion was even more obvious when cells were at the late exponential stage (Figure 4B). Therefore, X2 modules, especially the X2-C module in CipC, play an important role in cell adhesion to cellulose.

### X2 Modules Cannot Directly Bind to the Cell Surface of *Clostridium cellulolyticum*

To understand how X2 modules influence cell adhesion to cellulose, we performed *in vitro* X2 module-cell wall binding assay to test if the X2 modules mediate cellulosome localization on the cell surface. As ΔX2-C generated more significant physiological changes than ΔX2-N on cellulose, we expressed and purified X2-C module, CBM3a and ΔX2-C module (deletion of the conserved short motif) for *in vitro* assays (If not specified, we used X2 to represent X2-C in the following *in vitro* assays). In this binding assay, we separately mixed above-purified module proteins with *C. cellulolyticum* cells or *E. coli* (gram-negative) and *C. thermocellum* (Gram-positive) as controls. Western blotting did not detect the presence of X2 on any of these three strains post incubation with purified X2, indicating that X2 cannot directly bind to cell surfaces (Figure 4C). This agrees well with a recent study (Tarraran et al., 2021).

However, purified CBM3a was found in all three strains, indicating it is able to bind bacterial cell walls (Figure 4C). More interesting, we found a very weak signal of ΔX2 protein in the group of *C. thermocellum* and *C. cellulolyticum*. It is possible that ΔX2 could enhance the localization of cellulosomes on the surface of these cellulose degrading strains.

### X2 Module Cannot Directly Bind to Cellulose in *Clostridium cellulolyticum*

Since the X2 module could not directly bind to cell wall, here we performed *in vitro* X2 module-cellulose binding assays to test if X2 modules mediate the binding affinity between cellulosomes and cellulose. Using BSA as the negative control and purified CBM3a as the positive control for cellulose binding assays, we found that purified X2 only present in the supernatant instead of cellulose pellets, indicating that the X2 protein cannot directly bind to the cellulose (Figure 4D). Meanwhile, the ITC assay indicated that there was a weak interaction between the X2 module and the CBM3a (Supplementary Figure 4).

In summary, the X2 module in CipC cannot directly bind to cellulose or the bacterial cell surface. However, removing the conserved motif in the X2 modules decreased cellulose utilization and severely reduced cell attachment on cellulose.

## Discussion

The function of the X2 module has been studied for many years (Mosbah et al., 2000; Kosugi et al., 2004; Pasari et al., 2017), and almost all previous studies determining the function of the X2 module were based on *in vitro* biochemical assays. However, the *in vivo* function of the X2 module remains elusive. In *C. cellulolyticum*, two X2 modules are located in the *cipC* gene and the nucleotide sequence identity between them are very similar (65% for the pairwise nucleotide sequence identity). Due to limitations of traditional genome editing, it was very difficult to inactivate both of the X2 modules while maintaining the functional and structural integrity of the CipC scaffoldin protein. Fortunately, with the development and application of CRISPR-Cas9 based genome editing tools, we were able to generate both single and double deletions of two conserved sites in the X2 module. To our knowledge, at the genomic level determination of the functions of the X2 module has never been reported in any strain. A previous study, found that the disruption of the *cipC* gene in *C. cellulolyticum* hardly affected growth on soluble sugar but led to barely any growth on cellulose (Maamar et al., 2004). Compared to their study, we also found that the mutation of X2 modules did not influence growth on cellobiose (Figure 3A). Although, the mutation of the X2 modules led to a longer lag phase and decrease in cellulose degradation efficiency, growth on cellulose was not severely affected as was observed for the *cipC* deletion strain. Even the growth rate and maximum cell biomass between WT and ΔX2-NC were similar (Figure 3B). All of these data indicated that the deletion of the conserved motif in X2 modules did not affect the basic functions of the CipC and the functional loss of the X2 modules may directly influence certain functions of the cellulosome, as noted by the observed decrease in efficiency of the cellulosome in cellulose degradation.

Our microarray analysis confirmed the disruption of the X2 module in the *cipC* gene did not influence gene expression. On the other hand, the results of the adhesion assay indicated that the function of X2 modules was related to binding affinity between the cells and cellulose (Figures 4A,B). It is known that the cell surface cellulosome is the bridge for adhesion between the cells and cellulose (Gal et al., 1997; Schwarz, 2001), although the mechanism of the localization of the cellulosomes on the surface of *C. cellulolyticum* remains unclear (Desvaux, 2005). Therefore, the lower binding affinity between cells and cellulose caused by mutation of X2 modules should be attributed to the functional change of cellulosomes, which was consistent with our assumption.

From the cell adhesion assay (Figures 4,B), we hypothesized three possible mechanisms for X2 modules in regulating the binding affinity between cells and cellulose. Although previous studies indicated that the X2 module might directly bind to the cell wall (Kosugi et al., 2004), our *in vitro* protein-cell wall binding assay indicated that the X2 module protein could not directly bind to the cell wall (Figure 4C). This is consistent with a recent study that scaffoldins containing X2 domains derived from CpbA were not able to bind to the *L. lactis* surface (Tarraran et al., 2021). Based on structural analysis of the X2 modules (Mosbah et al., 2000), the surface of the X2 module is predominantly covered by hydrophilic amino acids and only contains a hydrophobic shallow groove, which could explain why the X2 module could not directly bind to the cell wall. Notably, the ΔX2 module protein had weak binding with the cell wall. This might promote the binding between the cellulosomes and cell surface, suggesting more numbers of cellulosomes might locate on the cell surface of the mutant strain. When the conserved motif (NGNT) was deleted, the hydrophobic shallow groove became wider as structure modeling indicated (Figure 2), which is more similar to typical CBM modules where most hydrophobic residues protrude outside (Luis et al., 2013; Pasari et al., 2017). As a result, this structural change might allow the ΔX2 module to weakly bind to the cell wall. This possible mechanism will be further investigated in our future work.

A previous study indicated that the localization of the cellulosomes (localized on the surface/free living) did not significantly influence the cellulose degradation efficiency of the cellulosomes (Xu et al., 2016). If the function of the X2 modules was only related to the localization of the cellulosomes, we would not expect to observe a significant decrease in cellulose degradation efficiency and release of soluble sugars in the ΔX2-NC strain compared to the WT (Figures 3C,D). Meanwhile, the HMMER analysis found that many X2 modules existed in free cellulases, which also indicates that the main function of X2 modules is likely unrelated to localization of cellulosomes. Meanwhile, we did not observe the X2 module from *C. cellulolyticum* could direcly bind to cellulose (Figure 4D). In CBM modules, most hydrophobic residues are on the protruded on the surface to promote carbohydrate polymer binding (Luis et al., 2013). In contrast, few hydrophobic residues are on the surface of the X2 module, and all the polar residues are exposed to the solvent (Mosbah et al., 2000), which indicated the reason why the X2 module could not directly bind to the cellulose. Therefore, the only possible reasonable explanation for the cell adhesion assay was that the X2 module cannot directly bind to the cellulose but may promote the binding between the cellulosomes and cellulose.

Given that the CBM3a module of the CipC scaffolding protein is for binding to the cellulose, we speculated that the *in vivo* function of the X2 module was realized by promoting the binding between CBM3a domain and cellulose. Our ITC assay also found there was a weak interaction between the X2 module and the CBM3a (Supplementary Figure 4), suggesting that the X2 module might interact with CBM3a and promote its binding function. In fact, previous *in vitro* assay had already found that the CBM3-X2 module had a better cellulose binding affinity to crystalline cellulose compared to CBM3 module alone (Pasari et al., 2017; Zhang et al., 2018). The processivity of *Cc*Cel9A mutant that lacks the CBMX2s was significantly lower compared to that of the wild-type *Cc*Cel9A, indicating the X2 module could indeed promote the binding between the CBM3 module and cellulose (Pasari et al., 2017; Zhang et al., 2018). In addition, the X2 module was thought to be associated with cellulase activity, which rendered the Cel9A cellulase from *L. phytofermentans* to be significantly more efficient on crystalline cellulose than any of the known cellulases from *C. cellulolyticum* (Ravachol et al., 2015). Some cellulase engineering studies also pointed out that the integration of the CBM with the X2 module into some cellulases enhanced avicelase activities, such as Cel48F and Cel9G (Mingardon et al., 2007; Vita et al., 2019). Taken together, these data may explain why we observed that the phenotype of WT grown on cellulose was better than ΔX2-NC and why the X2 modules are always next to the CBM3a modules in free cellulases.

## Conclusion

In summary, precise deletion of the NGNT conserved sequences of the X module was a useful strategy to carry out functional *in vivo* studies as this approach maintained the structural and functional integrity of the cellulosomes. This strategy can be applied to study the function of X2 modules or other interesting modules within certain proteins in other bacteria with similar cellulases/cellulosome-producing systems. We found that (i) the mutation of the X2 modules in *C. cellulolyticum* could indeed influence the cellulose utilization efficiency, and (ii) the *in vivo* function of the X2 module was determined to be associated with binding affinity between cells and cellulose. Given that the X2 modules are widely distributed in cellulolytic bacteria and play important roles in cellulose degradation, all of these findings provide new perspectives on engineering those potential CBP bacteria to improve their cellulose degradation efficiencies or modifying commercial cellulases to improve their hydrolysis efficiencies.

## Data Availability Statement

The original contributions presented in the study are included in the article/Supplementary Material, further inquiries can be directed to the corresponding authors.

## Author Contributions

TX, XT, and JZ designed the experiments. XT, TX, MK, and JL performed all the experiments. XT, JL, MK, and TX wrote the manuscript. MK and JZ edited the manuscript. All authors were given the opportunity to review the results and comment on the manuscript.

## Conflict of Interest

The authors declare that the research was conducted in the absence of any commercial or financial relationships that could be construed as a potential conflict of interest.

## Publisher’s Note

All claims expressed in this article are solely those of the authors and do not necessarily represent those of their affiliated organizations, or those of the publisher, the editors and the reviewers. Any product that may be evaluated in this article, or claim that may be made by its manufacturer, is not guaranteed or endorsed by the publisher.

## Footnotes

1
swissmodel.expasy.org

2
https://www.ebi.ac.uk/Tools/hmmer/
