# Supplementary material for: In vivo Functional Characterization of Hydrophilic X2 Modules in the Cellulosomal Scaffolding Protein
Source: Front Microbiol. 2022 Apr 7;13:861549. doi: 10.3389/fmicb.2022.861549 (PMC9022034; doi:10.3389/fmicb.2022.861549)
Supplement: Supplementary file 1 [file Image_1.pdf]

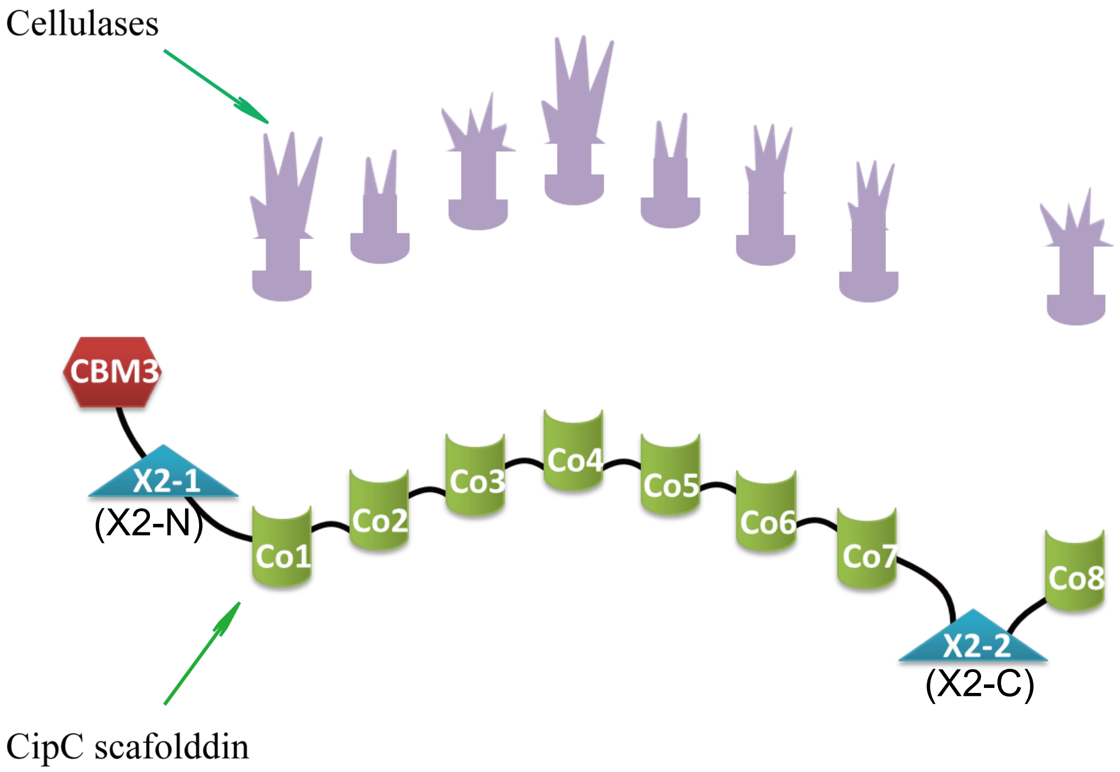

Figure S1. Scheme of the modular structure of the CipC scaffoldin in *C. cellulolyticum*. The CipC contains one carbohydrate binding module, eight cohesion modules and two X modules. The first X2 module (X2-1) was named as X2-N since it is located near the N terminus of CipC protein. The second X2 module (X2-2) was named as X2-C since it is located near the C terminus of CipC protein.
