# Supplementary figures and images for: In vivo Functional Characterization of Hydrophilic X2 Modules in the Cellulosomal Scaffolding Protein
Source: Front Microbiol. 2022 Apr 7;13:861549. doi: 10.3389/fmicb.2022.861549 (PMC9022034; doi:10.3389/fmicb.2022.861549)

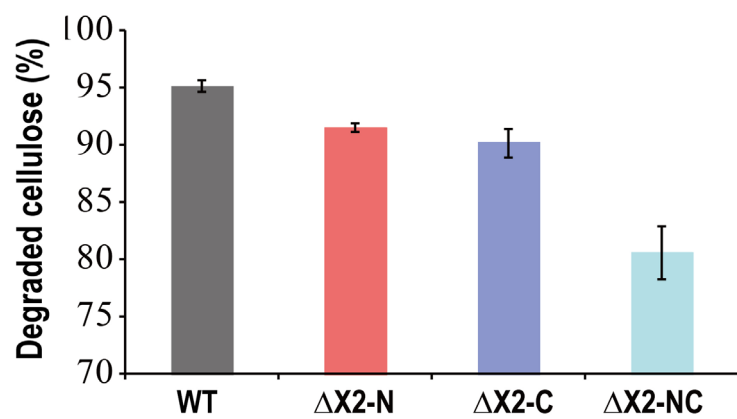

Figure S3. Degraded cellulose (%) for each strain at the final time point (260 h).

Supplement: Supplementary file 3 [file Image_3.pdf]
