# Supplementary material for: In vivo Functional Characterization of Hydrophilic X2 Modules in the Cellulosomal Scaffolding Protein
Source: Front Microbiol. 2022 Apr 7;13:861549. doi: 10.3389/fmicb.2022.861549 (PMC9022034; doi:10.3389/fmicb.2022.861549)
Supplement: Supplementary file 4 [file Image_4.pdf]

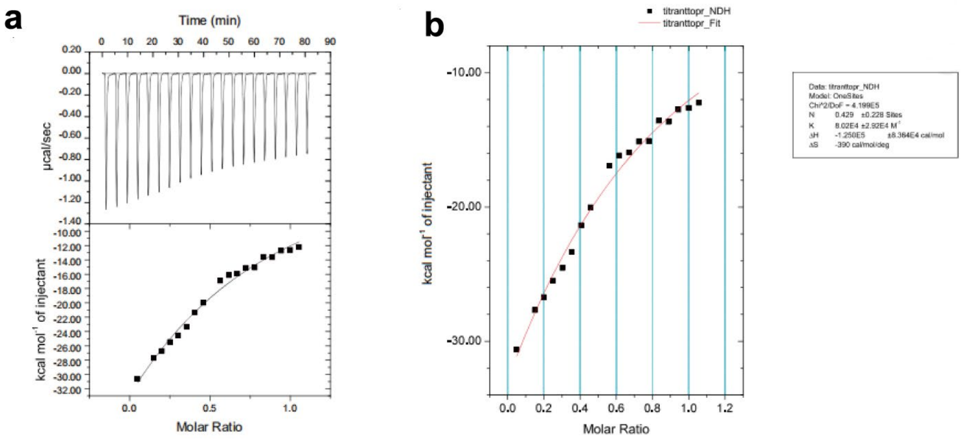

Figure S4. Isothermal titration calorimetry (ITC) data for binding interactions between CBM3a and the X2-C module. There is weak binding between them, and the binding affinity constant is $8.02 \pm 2.92 \times 10^4$ M^{-1}.
