# Supplementary material for: In vivo Functional Characterization of Hydrophilic X2 Modules in the Cellulosomal Scaffolding Protein
Source: Front Microbiol. 2022 Apr 7;13:861549. doi: 10.3389/fmicb.2022.861549 (PMC9022034; doi:10.3389/fmicb.2022.861549)
Supplement: Supplementary file 5 [file Table_1.pdf]

Table S1. The description of X2 modules aligned in Figure S2.

	organism	protein
WP_014313401.1:1109-1186	Clostridium sp. BNL1100	cellulose-binding domain-containing protein
WP_004622334.1:1201-1278	Ruminiclostridium papyrosolvens	cellulosome anchoring protein cohesin region
WP_080066193.1:1128-1205	Ruminiclostridium hungatei	cellulosome anchor protein
PYG86545.1:1168-1245	Ruminiclostridium sufflavum DSM 19573	cohesin domain-containing protein
EMS73510.1:1-66	Ruminiclostridium cellobioparum subsp. termitidis CT1112	cellulosome anchoring protein cohesin subunit
TFE82446.1:57-129	Paenibacillus sp. MEC069	hypothetical protein B5M42_25030
WP_185257592.1:829-911	Anaerocolumna sp. CTTW	xyloglucanase
WP_127531582.1:523-606	Paenibacillus kobensis	beta-mannosidase
WP_178943838.1:865-947	Paenibacillus curdlanolyticus	xyloglucanase
WP_013291799.1:1379-1449	Clostridium cellulovorans	cellulose-binding protein
WP_081756777.1:613-696	Gorillibacterium massiliense	beta-mannosidase
HAB60394.1:771-853	Lachnospiraceae bacterium	xyloglucanase
WP_184092446.1:649-730	Anaerocolumna cellulosilytica	glycoside hydrolase family 9 protein
WP_033165790.1:739-822	Clostridium sp. KNHs205	glycoside hydrolase family 9 protein
WP_103203479.1:669-751	Herbinix hemicellulosilytica	exoglucanase
HHT57230.1:198-274	Herbinix luporum	cellulosome anchor protein
WP_174818588.1:767-850	Paenibacillus kobensis	xyloglucanase
EMS73510.1:1-66	Ruminiclostridium cellobioparum subsp. termitidis CT1112	cellulosome anchoring protein cohesin subunit
