# Supplementary material for: In vivo Functional Characterization of Hydrophilic X2 Modules in the Cellulosomal Scaffolding Protein
Source: Front Microbiol. 2022 Apr 7;13:861549. doi: 10.3389/fmicb.2022.861549 (PMC9022034; doi:10.3389/fmicb.2022.861549)
Supplement: Supplementary file 2 [file Image_2.pdf]

```
                          1        10                  20             30                   40             50
X2_C                      TITPSTASFDKYVP..ANVNVTLTPNGNTFKGI....TGLTSGTDFTVSNNVVTISKSYL
WP_014313401.1:1109-      TITPTTASFDKYVP..ANVSVTLTPNGNTFKGI....TGLTSGTDYTVSNNVVTISKSYL
WP_004622334.1:1201-      TITPATASFDKYVP..ANVTVTLTPNGNTFKEI....TGLTSGTDYTVSGNVVTISKSYL
WP_080066193.1:1128-      SITPTTATFDKYVP..ADVAVTLTPNGNTFKGI....TGLTQGTDYTVSGNSVVILKGYL
PYG86545.1:1168-1245      TLSATTATFDKYAP..ADISVTYTANGNTFAGI....TGLTKGTDYTVSGSTVTISKSYL
QNU67288.1:2318-2394      .INPTSATFDKYAP..DDIKVTYSANGNTFKGI....TGLVSGTDYTVSGNTVTISKAYL
HHT57230.1:198-274        .ISPKSATFNKSAP..RNITVTLTPNGNTFNGI....SGLTRNTHYTVSGNTVTILSSYL
EMS73510.1:1-66           ............MP..ADVTVTLTPNGNTFKGI....TGLVQGTDYTVSGNTVVISKNYL
TFE82446.1:57-129         .ISPTTAAFDKKVSAQADVPITLTLNGNTFSGVWNGAAALTAGTDYTVAGNVVTLQKAYL
WP_185257592.1:829-9      .ISPTSAAFDKNTAKQADITVTISPNGNTLNGIKNGTTALTAGTDYTVSGNTVVIKKSYL
WP_127531582.1:523-6      TITPTSASFDKKTTAQADISVTMTLNGNTLNGVKKGTSSLVSGTDYTVSGTTVTIKKSYL
WP_081756777.1:613-6      TISPTSATFDKKTANQADIAVTMTLNGNTLSGIKNGSASLVSGTDYTVSGTTVTIKKSYL
WP_174818588.1:767-8      SITPASASFDKKTANQADISVALTLNGNTLSAIKNGSASLVSGTDYTVSGTTVTIKKSYL
WP_178943838.1:865-9      .ITPTSATFDKNTANQADIGVTLTLNGNTLSGIKNGSASLISGTDYTVSGSTVTIKKSYL
HAB60394.1:771-853        .ITPTSSSFDKNVTNQKDITVTMTLNGNTLNGITRGTNTLVSGTDYTVTGNVVTIKKEYL
WP_103203479.1:669-7      .ITPTSATFDKNEDNQADINVTVTYNGNTLVGIKSGDSYLTEGTDYTVNGNIVTIKKEFL
WP_033165790.1:739-8      TISITQAEFDKHTPNQQDIAVDITYNGNTLSGIRNGSTLLTSGTDYTLSGNTVTLLSSYL
WP_184092446.1:649-7      SITPVTAAFDKYDPK..DITVTVNYNGNTLNSIKNGTTTLVKGTDYTVTGDAIKLAASYL
WP_013291799.1:1379-      TVAPTAVTFDK..ANQADAAITMTLNGNTFSAIKNGTATLVKGTDYTVSENVVTISKAYL
X2_N                      TINPTSIS..AKAGSFADTKITLTPNGNTFNGI....SELQSSQYTKGTNEVTLLASYL
consensus>50              .itptsa.fdk..p.qa#i.!tlt.NGNTf.g!..g.t.ltsgtd%Tvsgnt!ti.ks%L

                              60        70
X2_C                      STLAVGSK.TLTFDFGVTN.NPVLTL
WP_014313401.1:1109-      STLAVGSK.ALTFDFGVTS.NPVLTL
WP_004622334.1:1201-      STLAVGSK.TLTFDFGVTN.NPVLTL
WP_080066193.1:1128-      SSQALGTK.ALTFDFGSTS.NPVITL
PYG86545.1:1168-1245      STLAVGTK.ALTFDFGTAS.NPVLTV
QNU67288.1:2318-2394      STLSLGTT.SLTFDFGSSR.NPVLTI
HHT57230.1:198-274        STLTLGTK.RLTFDFGVSN.NPVLNL
EMS73510.1:1-66           NTLAIGTK.TLTFDFGVTA.NPVLTL
TFE82446.1:57-129         ATLANGTA.TLVFKF...........
WP_185257592.1:829-9      SGRPVGTT.SLTFDFSAGS.DPVLSI
WP_127531582.1:523-6      AAQPVGTT.NLTFDFSAGT.DPVLTV
WP_081756777.1:613-6      AAQAVGTT.NLTFDFSAGN.DPVLAI
WP_174818588.1:767-8      AAQSVGTT.NLTFDFSAGT.DPVLAV
WP_178943838.1:865-9      ASLAVGMA.SLTFDFSAGI.DPVLSI
HAB60394.1:771-853        STLSTGNT.TLIFDFSAGS.DKNLTI
WP_103203479.1:669-7      AEQAVGSL.TLVFDFSAGL.DRTLTV
WP_033165790.1:739-8      ASKPLGKL.ELTFDFNKGN.DPVLTV
WP_184092446.1:649-7      STLTTGTT.KLVFDFSAGM.DPALTI
WP_013291799.1:1379-      AKQT.GTV.TLEFVF...........
X2_N                      NTLPENTTKTLTFDFGVGTKNPKLTI
consensus>50              stlavgt...LtFdF..g..npvlti
```

Figure S2. Alignment of the X2 modules among different species. The description of above aligned X2 modules is listed in Table S1.
